# Examining a brief web and longitudinal app-based intervention [Wysa] for mental health support in Singapore during the COVID-19 pandemic: mixed-methods retrospective observational study

**DOI:** 10.3389/fdgth.2024.1443598

**Published:** 2024-12-23

**Authors:** Chaitali Sinha, Dyuthi Dinesh, Creighton Heaukulani, Ye Sheng Phang

**Affiliations:** ^1^ Wysa, Boston, MA, United States; ^2^ MOH Office for Healthcare Transformation, Singapore, Singapore

**Keywords:** cognitive restructuring, attribution style, digital mental health, Singapore mental health, conversational agent (CA), Wysa, mindline, digital mental health intervention

## Abstract

The COVID-19 pandemic in Singapore led to limited access to mental health services, resulting in increased distress among the population. This study explores the potential benefits of offering a digital mental health intervention (DMHI), Wysa, as a brief and longitudinal intervention as part of the mindline.sg initiative launched by the MOH Office for Healthcare Transformation in Singapore. The paper aims to (i) Evaluate the engagement and retention of Singaporean users across the brief intervention on the mindline.sg website and the longitudinal app version of Wysa; (ii) Examine the types of negative thoughts and challenges managed during the pandemic; and (iii) Assess the impact of the conversational agent (CA) in supporting cognitive restructuring across attributional styles and cognitive patterns. A retrospective observational design with a mixed-methods approach was utilized. Website users (*N* = 69,055) and app users (*N* = 4,103) from September 1, 2020, to July 25, 2022, were included in the study. Engagement and retention were evaluated through usage data, and T-tests were used to compare engagement and retention between the app and website. A thematic analysis assessed the types of negative thoughts and the success of cognitive restructuring. Logistic regression was used to estimate the likelihood of restructuring based on negative thought type and attributional style. Users who used the longitudinal intervention after first using a brief intervention demonstrated significantly higher engagement and retention (*P* < 0.001). In user ratings received for mindline.sg (*n* = 8,959), 83.03% rated the app 3 or higher (out of 5) on helpfulness. 91.6% of the users (*n* = 862) who attempted cognitive restructuring (*n* = 790) on the app successfully reframed a thought. A single conversation with Wysa was also significantly associated with the ability to restructure future-oriented negative thoughts (*P* < 0.001) and internal, stable and global (*P* < 0.001) negative thoughts, while other attributional styles required more intervention. Psychosocial challenges managed by users during COVID-19 were also documented through negative thoughts mentioned within the CA. The findings demonstrate that brief interventions can facilitate enhanced engagement with DMHIs and that digital interventions can successfully facilitate cognitive restructuring and improve mental health outcomes. The study provides useful inputs to guide the development of DMHIs and improve their effectiveness.

## Introduction

The early months of the COVID-19 pandemic were marked with distress from the uncertainty and unpredictability surrounding the virus, and saw an increased prevalence of mental health concerns in the global population (1, 2). Increasing mortality rates, active cases, continued media coverage, and isolating health protective measures contributed to increasing levels of distress (3–7). This time period was associated with a spike in depression, anxiety, negative thoughts, and burnout (8). Global public health challenges included the increased psychosocial needs of the population, with service availability and continuity being compromised by isolation and mitigation measures aimed at preventing and controlling the spread of the epidemic (9). With mental health services disrupted, telehealth and digital mental health interventions (DMHIs) were used to tackle the surge of mental health concerns and symptoms in communities (10, 11).

Cognitive-behavioral models (12–14) have demonstrated significant positive correlations between mental health concerns such as anxiety and depression, maladaptive schemas (15) and cognitive distortions (16–18). These can often be indicative of the depth and nature of distress, and also clarify interventive pathways (19–21). This has proven useful in the design of brief interventions, where the targeting of core beliefs and attributional styles has led to successful cognitive restructuring and the reduction of distress (22).

Brief interventions delivered through DMHIs have become a sustainable way to address gaps in mental health service provision as they are able to leverage everyday technology (23), and offer it at scale. The anonymity, accessibility, ability to surpass social distancing, and support overburdened clinicians all add to the value of DMHIs as they circumvent structural and attitudinal barriers to accessing usual care (23, 24).

Singapore is among the countries that embraced the use of accessible digital mental health interventions in their healthcare system (25, 26). The Ministry of Health (MOH) launched a COVID-19 Mental Health Taskforce (CoMWT) that reviewed the psychosocial impact of the pandemic on the country's population and provided recommendations to address the mental health concerns of its citizens. The recommendations noted severe burnout, symptoms of anxiety and depression (27–29) in response to isolation, increased workload and financial stress (8). In June 2020, the Singaporean government launched mindline.sg—a stress management and coping website that consolidated access to local resources including a web version of Wysa that could offer brief, guided interventions. Wysa is an AI-led, cognitive behavioral therapy (CBT) digital mental health conversational agent that has proven to be feasible (30), acceptable across global audiences, and clinically efficacious, supported by its unique ability to build a therapeutic alliance with users (31) which ensures high retention and engagement (32).

This study utilized Wysa, to evaluate its engagement as a public health service for Singaporean citizens during the COVID-19 pandemic. The study has 3 objectives: (1) to evaluate the engagement and retention patterns of Singapore users across the brief intervention (website) and the longitudinal (app) version of Wysa; (2) to examine the types of negative thoughts and challenges being managed during the pandemic; (3) to evaluate the impact of AI-led support and its ability to support cognitive restructuring across attributional styles, and cognitive patterns.

## Methods

### Setting

In response to the COVID-19 pandemic, Singapore's MOH Office for Healthcare Transformation, a part of the Ministry of Health, launched a website (mindline.sg) for all users in Singapore, providing various resources on mental health in June 2020. The goal of this website was to provide an anonymous and trusted platform for mental health resources. Wysa, an AI-led digital mental health conversational agent (CA), was provided on the website to enable mental health support for users. It provided users in Singapore with free access to a wide range of self-assessment and self-management exercises. The users could also download the Wysa app from within the brief intervention on the website.

### Study design

This brief intervention was made available to all citizens and residents by the Singapore Ministry of Health and Transportation through its portal mindline.sg as a public health intervention during the COVID-19 first wave in the region. During the study period of Sept 1, 2020 to July 25, 2022, 69,055 unique users visited Wysa through the mindline.sg website, and 4,103 users downloaded the mobile app version. This study utilized a retrospective observational design, with a mixed-methods approach to examine the usage and affective data from the public health rollout between this time period across the app and the mindline.sg website.

### Intervention

Wysa is a conversational agent (CA) which delivers CBT-based interventions. It listens and responds to the user's distress by recommending techniques and self-care tools based on CBT, behavioral reinforcement, and mindfulness, among other evidence-based therapies. The app does not incorporate any gamification elements in its therapeutic interventions and walks the user through a therapeutic dialogue intended to prompt self-examination and finding a more balanced thought.

Wysa uses proprietary AI and Natural Language Processing/Understanding (NLP/NLU) algorithms that classify user inputs to understand messages and guide conversations. The AI operates within a decision-tree framework, using fixed, pre-defined scripts validated by clinicians for safety. This rules-based approach ensures that all responses are predictable and clinically safe. The responses within the conversational agent (CA) are written and validated by a team of clinicians.

Users coming through the mindline.sg website would first access a web version of Wysa built for mindline.sg. This was integrated directly within the main mindline.sg website, through which a user could access a full-fledged interaction with Wysa on the web. The AI-led conversational agent (CA) guided users through brief interventions that could be completed within 5–10-minute interactions.

The user could also click a “Download App” button at the bottom of this web page which would allow them to access the Wysa app with the ability to converse with the conversational agent, and all of the 150 + interventions within its self-care library.

### Ethical considerations

mindline.sg and the Wysa mobile app ensured user anonymity by not requiring any personal information to access or during use. Only user nicknames were requested to personalize the conversational experience.

To maintain user anonymity and to be consistent with Wysa's confidentiality protocols, demographic data and personally identifiable information were not collected within the mobile app. User data were adequately secured according to the organization's privacy, security, and safety policies. As the study involved analyzing real-world data from an anonymous nonclinical population, it was exempt from registration in a public trial registry (according to OHRP guidelines) (33). The users voluntarily downloaded the app after having consented to the app's Terms of Service and Privacy Policy. Given the anonymous and aggregated nature of the study, there was no compensation or monetary incentive involved.

For ethical and privacy reasons, the authors did not have access to continuous user messages on the mindline.sg platform and on the Wysa app. Only minimal and limited conversational data extracted based on keywords were used for this research, and no longitudinal data was used. The study data set was de-identified using one-way cryptographic functions. User data were adequately secured according to the organization's privacy, security, and safety policies. Wysa is GDPR and HIPAA compliant ensuring robust data protection.

To address potential bias in analysis, the authors from each institution independently analyzed the data presented. No generative AI was used in any portion of the manuscript writing.

### Measures

The following measures and data types were used: (1) usage data within the app (2) textual snippets from users. The data included within the manuscript was cleaned to ensure that it is relevant and valid data (from the chosen time period, geography, mindline.sg users, removal of duplication).

### Analysis

#### Objective 1: to evaluate the engagement and retention of Singaporean users across the brief intervention on the mindline.sg website and the longitudinal app version of Wysa

Usage data from all users (*n* = 69,055) who utilized the brief intervention on either the mindline.sg website or the Wysa app was analyzed. The engagement and retention of users was separated on the basis of their modality: on Wysa for mindline.sg (the web version), or on the Wysa app. This analysis is representative of all users who utilized the web or app version of Wysa.

Engagement was calculated using the number of sessions completed, and retention was calculated as retention duration (i.e., users' last day of engagement with the app during the study period). Frequency of the use of self-guided interventions were also documented to further understand the needs of the population. The sessions were only counted toward engagement and retention if a user initiated a conversation with the CA, or an intervention (tool) within the app. Passive events, such as opening the app, were not counted. All engagement and retention data was collected automatically via the app's usage log.

An indicator for the helpfulness of the app was aggregated through self-reported user responses to a 5-point feedback scale which was offered at the end of the intervention.

Engagement and retention patterns were compared between users who downloaded the Wysa app after the brief intervention on the mindline.sg website, and users who directly used the Wysa app without any brief interventions. App users who underwent a brief intervention on the mindline.sg website were compared to that of 1,000 iterative samples of app users who did not undergo any brief intervention in the same time period.

#### Objective 2: to examine the types of negative thoughts and challenges managed during the pandemic

A sub-sample of users (*n* = 862) has been analyzed for the purpose of this objective, with only those users included who explicitly linked their affective state to the pandemic and its related impact.

This study only uses textual snippets to maximize privacy and anonymity of any conversation within the app. The terms used to capture users' affective states related to COVID-19 were restricted to keyword extractions of the following terms: “quarantine, lockdown, pandemic, health, health anxiety, vaccine, covid, and corona.” To identify user messages containing emotions, keyword identification and contextual snippet extraction was conducted. Textual snippets that had at least one or more of the pandemic-related keywords and emotion words were included within the dataset, and the examined thoughts were extracted from a specific query about current negative thoughts.

The negative affective states and thoughts of the users were examined from the extracted snippets using Beck's cognitive triad model, which segregated the snippets into the domains based on cognitive patterns related to the self, the world and the future (12). The snippets within the cognitive triad categories, were further split into the three levels of the hierarchical organization of thinking, namely core beliefs, intermediate beliefs and negative automatic thoughts (12).

In our analysis of the data, we used a combination of inductive and deductive thematic analysis (34) to analyze perceived needs and experiences exhibited by user's expressed emotions. The analysis began with the authors reading all conversational snippets several times to familiarize themselves with the data. The snippets were identified and categorized into the cognitive triad categories, and levels of thinking and themes related to affective states were grouped and identified.

#### Objective 3: to assess the impact of the conversational agent (CA) in supporting cognitive restructuring across attributional styles, and cognitive patterns

For evaluating the negative thought patterns and restructured thoughts during the pandemic, singular textual snippets of the negative thought and the stated resolution by the user were extracted at a singular conversational nodal point to maximize conversational privacy.

In the third objective, the negative thoughts stated by the user in Objective 2, and their corresponding successful or unsuccessful resolutions, were examined. The categorized negative and the final restructured thoughts were further split based on attributional styles using established frameworks (14, 35). As per the model, the users' negative statements were categorized in a matrix of three dimensions: (1) internal or external: based on the perception of who caused the event; (2) stable or unstable: based on the perceived permanence of the situation; and (3) global or specific: based on the perceived pervasiveness of the situation. The matrix was examined to determine the changes in attributional styles between the original negative thought and the restructured one.

### Regression analyses

The data from objectives two and three are examined in statistical analyses to discover potential factors amongst negative thought patterns and their attributional style that are associated with the success of restructuring the thought.

We perform a multinomial regression with negative thought domain as the independent variable and restructuring success as the dependent variable. We also perform a separate multinomial regression with attributional style from the cognitive triad as the independent variable and restructuring success as the dependent variable. We do not expect there to be meaningful interactions between negative thought domain and attributional style under the triad, and so we do not explore interactions amongst these classifications. Finally, we explore whether specific changes from one style to another (or a lack of change) is associated with restructuring success by analyzing analogous multinomial regression models, where the covariates are all possible changes in thought style. In particular, in the model for negative thought domains, there are nine possible changes in domain, i.e., self to self, self to world, self to future, etc. In the model for cognitive triad attributional style, there are three different independent variables, each with four possible changes in attributional style. For example, for the independent variable on who caused the event (cf. Objective 3), the possible changes (or lack thereof) are “internal to internal”, “nternal to external”, “external to internal”, and “external to external”. We report the coefficients of each regression and highlight significance at the 0.05 level.

## Results

### Objective 1: to evaluate the engagement and retention of Singaporean users across the brief intervention on the mindline.sg website and the longitudinal app version of Wysa

#### Website: Wysa on mindline.sg

During the study period, the Wysa on mindline.sg website had 69,055 unique users, who completed 96,856 sessions with the CA. 83.03% of user ratings (*n* = 8,959) were “Good” or “Very Good” with a 3+ (out of 5) rating on helpfulness. The mean number of messages exchanged within the brief intervention were 5.70 (SD 8.44).

The most used interventions on the mindline website were Energy and Mood Check-in (64.86%, *n* = 62,822), Cognitive Restructuring (9.89%, *n* = 9,580), Mindfulness (9.02%, *n* = 8,732), Motivational Story (6.24%, *n* = 6,045), Gratitude (5.29%, *n* = 3,322), Acceptance (2.60%, *n* = 2,518), Thought Recording (2.12%, *n* = 2,050), and Sleep (1.85%, *n* = 1,787).

In the examination of the interventions most commonly used just before downloading the longitudinal app intervention, 18.49% of users downloaded after using a mindfulness intervention (*n* = 5,225), 10.84% after using a sleep intervention (*n* = 3,065), and 8.89% after thought recording (*n* = 2,512).

#### Wysa app

##### mindline.sg Website users on the Wysa app

During the study period, the Wysa app was used by 4,103 users within this cohort. The users utilized 48,932 sessions, with a mean utilization of 10.78 (SD 22.49) sessions per user, and a mean retention of 61.60 days (SD 109.11).

The most used interventions on the app were Mood Check-ins (46.03%, *n* = 22,525), Cognitive Restructuring (19.62%, *n* = 9,600), Mindfulness (17.57%, *n* = 8,597), Gratitude (7.39%, *n* = 3,619), and Thought Recording (4.18%, *n* = 2,045).

##### Wysa app users: after brief intervention on mindline.sg vs. no brief intervention

In the comparison of app users who underwent a brief intervention on the mindline.sg website, and of 1,000 iterative samples of app users who did not undergo any brief intervention in the same time period, users who utilized a brief intervention before using the app, used a greater number of sessions on the app (Table 1) (*P* < 0.001), had a longer retention period (more active days) (*P* < 0.001), and a larger total interval of engagement (*P* < 0.001) than those who did not utilize a brief intervention before app usage. Those without a brief intervention utilized a longer length of session than brief intervention users (*P* < 0.001).

**Table 1:** Comparison of engagement and retention between users who were delivered a brief intervention before app usage and users who did not use a brief intervention before app usage.

	Brief intervention users	Non-brief intervention user sample 1	Non-brief intervention user sample 2	Non-brief intervention user sample 3
Number of sessions				
Mean	13.80	4.44	4.47	5.21
Median	6	2	2	2
Mann-Whitney W	N/A^a^	5,60,227	5,69,817	5,42,331
*P* value	N/A	*P* < 0.0001	*P* < 0.0001	*P* < 0.0001
VDA estimate^b^	N/A	0.28^c^	0.28^c^	0.28^c^
Total active days				
Mean	7.79	2.41	2.48	2.84
Median	3	1	1	1
Mann-Whitney W	N/A	5,63,677	5,77,340	5,50,728
*P* value	N/A	*P* < 0.0001	*P* < 0.0001	*P* < 0.0001
VDA estimate	N/A	0.29^c^	0.28^c^	0.29^c^
Total interval of engagement				
Mean	76.76	26.20	33.08	28.41
Median	13	0	0	0
Mann-Whitney W	N/A	5,51,589	5,61,580	5,42,018
*P* value	N/A	*P* < 0.0001	*P* < 0.0001	*P* < 0.0001
VDA estimate	N/A	0.30^c^	0.29^c^	0.29^c^
Session length				
Mean	8.20	10.34	11.12	10.69
Median	4	6	7	6
Mann-Whitney W	N/A	2,20,14,364	2,18,01,135	2,44,44,944
*P* value	N/A	*P* < 0.0001	*P* < 0.0001	*P* < 0.0001
VDA estimate	N/A	0.28^c^	0.29^c^	0.29^c^

^a^ N/A, not applicable.

^b^ Effect size.

^c^ Medium effect size.

### Objective 2: to examine the types of negative thoughts and challenges managed during the pandemic

The examined negative thoughts were split into the three categories of the cognitive triad: the self, the future and the world.

#### The self

In thoughts that were categorized as “negative about self”, the most commonly reported affective state was that of sadness. This consisted of related terms such as fatigue, grief, disappointment and anguish.

Most self-oriented negative automatic thoughts were triggered by not being able to fulfill the needs of those around them, “disappointing people” or not “being able to care for people.” Some mentioned thoughts related to not feeling “competent” or “being the worst” or feeling evaluated by those around them, and some stated thoughts related to the anxiety of what they haven't been able to do, or what was expected of them.

In thoughts classified as intermediate beliefs, most were commonly linked to a sense of worth or achievement including “If I were better, things like this would not have happened” or needing to be “more competent”, have “more achievements”, “more tolerant”, “more competent.” Some users also mentioned linkages between fulfilling responsibilities and self-worth stating they were “entirely responsible for everything.” Most users who directly stated a core belief mentioned worthlessness or helplessness.

#### The world

With respect to negative views of the world, the most reported affective state was that of sadness which consisted of feelings of unhappiness, depression, grief and disappointment.

Most world-oriented negative automatic thoughts were triggered by rejection. They expressed “my friends don't like me” or that “others would judge me and see me as weird.” Automatic thoughts also revolved around feeling mistreated: “I feel so neglected as my colleagues don't treat me as part of their teams”, “People take advantage of me despite me trying to help them.” Interpersonal conflict was also a common theme. Users mentioned that they “felt bullied” and like others were “picking fights” with them. Some users also expressed guilt and concern for others. They reported feeling like they “might be the reason” a loved one was facing difficulty, and that they “didn't know how to love” their partner. Few users also expressed not having their expectations met. They were disappointed in others not “owning up to any responsibility” in their interpersonal conflict, and in others not being able to “ accept their mistake”.

Most world-related intermediate beliefs expressed rule-based beliefs related to a sense of duty. They felt strongly against individuals “who exploit people” and who are “not cooperative”. Users expected others to reciprocate efforts with statements like “I worked hard and she should too.” Moreover, users had a sense of responsibility. They reported feeling like a “bad partner” and “can't make him happy.” They felt like mistakes were their “fault: I should have checked with my supervisor.”

The core beliefs were associated with the world being harsh and distrustful. Users feared that their partner was betraying their trust because they are “not enough”. They also felt like they could not trust their family (“dad can't be trusted”) and that people “don't care about other people.” Some users expressed feeling unlovable by others, “there's something fundamentally wrong with me.”

#### The future

When writing about their negative thoughts about the future, most users mentioned affective states related to anxiety. Users reported feeling fear, apprehension, stress, worry, nervous, overwhelmed, overburdened, insecure, helpless and dissatisfied.

In thoughts related to the future, most automatic thoughts were in response to situational causes like time management, dealing with work pressures, struggling to “meet the deadline” and “finishing everything on time.” Most users also reported worries about performance, about “wanting to do well” at school and in examinations, fear of failure and worries about outcomes. Some users mentioned financial concerns (for example: “need to have a higher paying job” or “No money: how to survive”) and medical concerns, like “stress about possible surgery”, illness and/or hospitalization. Few users mentioned job search, dissatisfaction with work, desire to quit, unemployment, relationship troubles, daily domestic stress, and physiological symptoms of anxiety (for example, worry about “another panic attack”) as causes for negative affective states.

Most users expressed intermediate beliefs that were concerned with opinions about others and performance. Expressed concerns included: “I'm scared they won't understand and will just make fun of me again or they'll apologize without really understanding fully” or “I will try harder tomorrow but I'm not sure if my body can last.” Some intermediate beliefs had to do with work style and perfectionism. For example, “The only way to get the task done is to work overtime” and “I feel like I have to be perfect if not things will fall apart.”

**Figure 1:**
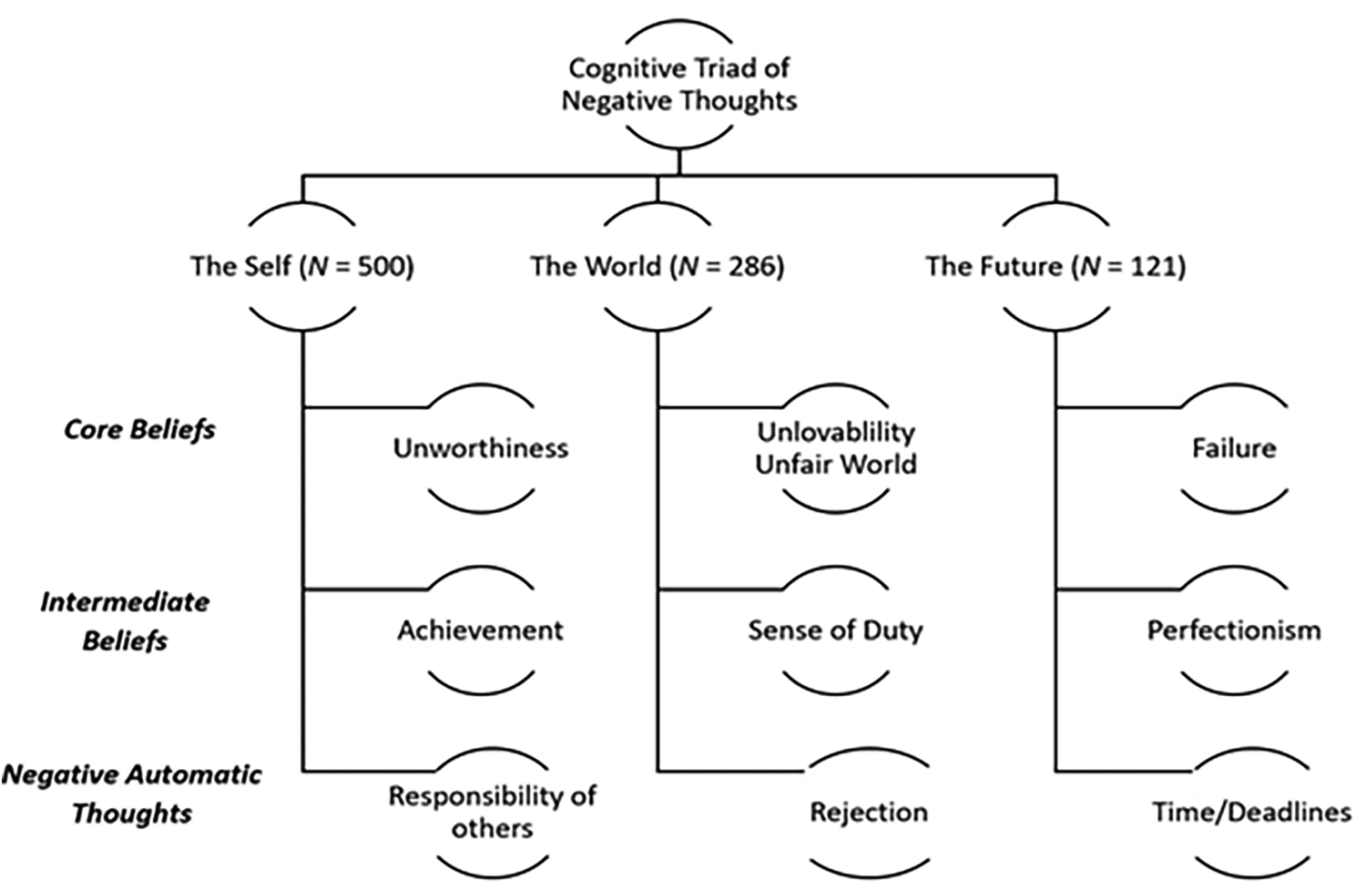
Themes within the hierarchical organization of thinking and Beck's cognitive triad.

Most users expressed core beliefs that were concerned with fear of failure. These fears mainly consisted of the following: “causing trouble to people”, “letting people down”, “bad habits”, job loss, stepping out of “comfort zone”, investments and family well-being (Figure 1).

### Objective 3: to assess the impact of the conversational agent (CA) in supporting cognitive restructuring across attributional styles, and cognitive patterns

In a sampling (*n* = 862) of those who utilized cognitive restructuring on Wysa, 91.6% (*n* = 790) had a successful CBT breakthrough. 66.6% (*n* = 574) successfully restructured the thought in their first attempt, while the remaining needed further support and exemplification for successful restructuring. Most negative thoughts were internal, stable and global (*n* = 219) in their attribution, and were most commonly restructured into internal, unstable and specific (*n* = 210) attributions. The most common restructuring was the transition from a stable, global attributional style to an unstable attributional style (*n* = 414) (e.g., “I feel that my friends don't like me” restructured to “I can work hard to be a better person and make friends who appreciate me”). Table 2 shows the observed transitions in Attributional Style from Negative to Restructured Thoughts.

**Table 2:** Transitions in attributional style from negative to restructured thoughts.

Initial negative attribution		Final restructured attribution	Frequency
Internal	→	External	27
External	→	Internal	128
Stable	→	Unstable	414
Unstable	→	Stable	45
Global	→	Specific	149
Specific	→	Global	55

#### The self

##### Negative thoughts

In negative thoughts related to the self, the most common attributional style was (1) internal, stable and global (*n* = 198). Many stated, “I am not good enough”, “The thought is that I'm a failure.” (2) Internal, stable and specific (*n* = 40) where users had negative thoughts about themselves in specific situations. One said, “I'm not doing enough for exams”. Another user expressed regret by saying, “I lost him because of my …codependency.” For negative thoughts, when it came to individual attributional styles along each dimension, internal (*n* = 254), stable (*n* = 280) and global (*n* = 236) were the most common.

##### Restructuring

Internal, stable and global negative thoughts, were most commonly restructured into internal, unstable and specific (*n* = 82). Thoughts of not being good enough were replaced with thoughts like, “I am empathetic and thoughtful so I really want my colleagues and team to be happy and do well.” Users reassured themselves and restructured thoughts of unemployability as, “I have taken active steps to make myself employable. I may not always achieve what I want: but I can be kind to myself.” The above concern about being a failure was restructured as, “I am a hardworking individual so I would like to have grades which reflect my efforts.”

Similarly, internal, stable and specific thoughts were most restructured into the internal, unstable and specific (*n* = 24) category. For example, the above concern about exams was restructured as “I will try my best to achieve an ideal me tomorrow.” Additionally, the user who viewed themself as codependent felt reassured after stating, “Having an active self-awareness and eventually having a successful relationship” Here, while users' locus of control remained on themselves, they were able to shift stable and global negative thoughts to unstable and specific thoughts. For restructured thoughts, the most prominent attributional styles along each dimension were internal (*n* = 274), unstable (*n* = 262), and global (*n* = 148).

#### The world

##### Negative thoughts

When it came to negative thoughts about the world, the most common attributional styles were: (1) External, stable and global (*n* = 46), where users expressed concerns about others' overall behavior, “She was just nitpicking on us”, “He doesn't compliment me enough.” (2) External, stable and specific (*n* = 40) where users were concerned about other people's behavior in specific instances, “What if she stops being my friend.” or “sadness over the fact that he doesn't feel the same.”

For negative thoughts, when it came to individual attributional styles along each dimension, external (*n* = 96), stable (*n* = 105) and specific (*n* = 67) were the most common.

##### Restructuring

For external, stable and global negative thoughts, the most common restructuring was into internal, unstable and specific (*n* = 19). For example, a concern about nitpicking was restructured as “When others are behaving badly, I will keep in mind that it is not about me..”

Similarly, external, stable and specific thoughts were most often restructured into the internal, unstable and specific (*n* = 17) category and the internal unstable and global (*n* = 10). For example, a restructured thought stated “I am kind and thoughtful: so I really want to have a successful relationship.” Users shifted their locus of control onto themselves and acknowledged the temporal and specific nature of their original negative thought.

For restructured thoughts, the most prominent attributional styles along each dimension were internal (*n* = 102), unstable (*n* = 103), and specific (*n* = 72).

#### The future

##### Negative thoughts

In negative thoughts about the future, the two most common groupings of attributional styles were: (1) internal, stable and specific (*n* = 17) where users shared their anxiety and fear about the future. For example, “What if I don't get a house that I want and save enough money for it?” or “I'm really stressed right now because it's submission week in a few days. There are so many submissions: there is a presentation: I have to design posters and slides. On top of that: I have to do other regular assignments. The workload is causing me to procrastinate a lot which in turn is causing me to panic more.” (2) Internal, stable and global (*n* = 13) here users shared a more general uncertainty related to the future stating, “I don't know what my future will be like” or expressed fear of failure as evidenced by: “Thinking that I won't be successful in the future.”

##### Restructuring

Negative thoughts about the future were mostly restructured into the “internal, unstable and specific” (*n* = 129) category. The users expressed a self-focused, possible solution, which targeted a specific area of their lives and marked a break from stable thinking, for example: “I can do what I put my mind to. I've been conscientious and hard working thus far: I shouldn't give up now and still press on to deliver quality work for my homework now.”

In examining the transition between negative to restructured thoughts, the most common and evident shift was from “internal, stable and specific” to “internal, unstable and specific.” For example, as mentioned above, the negative thought (“What if I don't get a house that I want and save enough money for it?”) was restructured into “I do still have some savings. I just need to be patient and work on it and yes, I will eventually get a house.” Similarly, “internal, stable and global” categorized negative thoughts were restructured into internal, unstable and specific. For example, the negative thought, “I need to do okay in school,” was restructured into “I am responsible and diligent: so I want to manage my studies well.”

#### Regression analysis

In Table 3, we report the regression coefficients in the multinomial regression models investigating if negative thought domains and attributional styles are associated with restructuring success. We find that the log-odds of restructuring the thought on the first attempt (compared to not successfully restructuring the thought) is 2.50 (*P* < 0.001), when the negative thought is about the future. No other terms in the model for negative thought domains are significant at the 0.05 level.

**Table 3:** Multinomial regression coefficient estimates investigating if negative thought domains and attributional style is associated with restructuring success.

	Restructured 1st attempt	Restructured 2nd attempt	Acceptance
Negative thought style			
Future (baseline)	2.50 (*P* < 0.001)*	0.39 (*P* = 0.42)	−3.39 (*P* = 0.10)
Self	−0.45 (*P* = 0.27)	0.03 (*P* = 0.95)	0.74 (*P* = 0.73)
World	−0.19 (*P* = 0.68)	0.64 (*P* = 0.25)	1.89 (*P* = 0.37)
Causal attribution			
Internal (baseline)	2.04 (*P* < 0.001)*	0.35 (*P* = 0.04)*	−2.33 (*P* < 0.001)*
External	0.35 (*P* = 0.20)	0.70 (*P* = 0.02)*	0.02 (*P* = 0.99)
Permanence attribution			
Stable (baseline)	2.19 (*P* < 0.001)*	0.56 (*P* < 0.001)*	−2.16 (*P* < 0.001)*
Unstable	−0.40 (*P* = 0.27)	0.26 (*P* = 0.53)	−68.05 (*P* = 0.99)
Pervasiveness attribution			
Specific (baseline)	2.49 (*P* < 0.001)*	1.32 (*P* < 0.001)*	−1.54 (*P* = 0.01)*
Global	−0.48 (*P* = 0.08)	−1.13 (*P* < 0.001)*	−1.19 (*P* = 0.12)

In all models, the baseline condition is a negative thought that was not successfully restructured, with style or attribution noted in the description of the constant term. The regression coefficient is displayed with its *P*-value in parentheses.

* Significance at the 0.05 level is highlighted.

In the model for causal attribution of the thought, restructuring a thought with a perceived internal cause on the first and second attempts were both significantly more likely than not restructuring, while acceptance was significantly less likely than not restructuring. It was also found that an external attributional style had a 0.70 (*P* = 0.02) log-odds of restructuring the thought on the second attempt, compared to not restructuring the thought.

In examining the permanence attribution of the thought, restructuring a thought with a perceived stable permanence on the first and second attempts were both significantly more likely than not restructuring.

In the model for pervasiveness in attribution of the thought, restructuring a thought with a perceived specific pervasiveness on the first and second attempts were both significantly more likely than not restructuring. It was also found that thoughts with a perceived global pervasiveness had a −1.13 (*P* < 0.001) log-odds of restructuring the thought on the second attempt, compared to not restructuring the thought.

In Table 4, the multinomial regression coefficients are displayed for the models investigating if changes in attributional style are associated with restructuring success. Considering the perceived cause of the negative thought, the log-odds of restructuring the thought on the 1st (log-odds: 2.71; *P* < 0.001) and 2nd (log-odds: 1.61; *P* = 0.04) attempts was more likely than not successfully restructuring the thought when the individual attributes the thought to an External cause and that attribution doesn't change. No other factor is significant at the 0.05 level in this model.

**Table 4:** Multinomial regression coefficient estimates investigating if changes in negative thought attributions (or lack thereof) are associated with restructuring success.

	Restructured 1st attempt	Restructured 2nd attempt	Acceptance
Change in causal attribution			
Constant (baseline: external, no change)	2.71 (*P* < 0.001)*	1.61 (*P* = 0.04)*	−9.27 (*P* = 0.90)
External to internal	−0.35 (*P* = 0.65)	−0.63 (*P* = 0.45)	7.08 (*P* = 0.92)
Internal to external	−1.48 (*P* = 0.07)	−1.61 (*P* = 0.07)	−4.47 (*P* = 0.99)
Internal, no change	−0.57 (*P* = 0.45)	−1.20 (*P* = 0.13)	7.11 (*P* = 0.92)
Change in permanence attribution			
Constant (baseline: stable, no change)	1.03 (*P* < 0.001)*	−0.51 (*P* = 0.16)	−1.61 (*P* = 0.003)*
Stable to unstable	1.39 (*P* < 0.001)*	1.28 (*P* = 0.001)*	−0.96 (*P* = 0.21)
Unstable, no change	0.45 (*P* = 0.30)	1.30 (*P* = 0.01)*	−10.23 (*P* = 0.93)
Unstable to stable	11.32 (*P* = 0.93)	−1.82 (*P* = 0.99)	−0.97 (*P* = 0.99)
Change in pervasiveness attribution			
Constant (baseline: global, no change)	1.60 (*P* < 0.001)*	−0.02 (*P* = 0.91)	−2.49 (*P* < 0.001)*
Global to specific	0.98 (*P* < 0.001)*	0.64 (*P* = 0.07)	−34.79 (*P* = 0.99)
Specific to global	0.43 (*P* = 0.25)	0.81 (*P* = 0.06)	−54.54 (*P* = 0.99)
Specific, no change	1.35 (*P* = 0.001)*	1.80 (*P* < 0.001)*	1.82 (*P* = 0.03)*

Displayed is the regression coefficient with its *P*-value in parentheses.

* Significance at the 0.05 level is highlighted.

Considering the perceived permanence of the situation regarding the negative thought, thoughts perceived to have either a stable permanence, regardless of whether that attribution changes, were more likely to be easily restructured (No change: log-odds = 2.71, *P* < 0.001; Change to unstable: log-odds = 1.39, *P* < 0.001). Thoughts that changed attribution from stable to unstable permanence (log-odds = 1.28, *P* = 0.001) or were unstable with no change (log-odds = 1.30, *P* = 0.01) were more likely to be restructured only on the second attempt, compared to not successfully restructuring the thought. Finally, a stable attribution that did not change was less likely to fall into the Acceptance category, compared to not successfully restructuring the thought (log-odds = −1.61, *P* = 0.003).

Considering the perceived pervasiveness of the negative thought, global attributions that do not change, are more likely to be restructured on the 1st attempt (log-odds = 1.60; *P* < 0.001) and less likely to fall into acceptance (log-odds = −1.61; *P* = 0.003), compared to not restructuring the thought. Thoughts with an attribution that changes from global to specific pervasiveness were more likely to be restructured on the 1st attempt (log-odds = 0.98; *P* < 0.001) compared to not restructuring. Finally, thoughts with a specific pervasiveness that don't change were less likely to not be restructured compared to all other outcomes.

## Discussion

The present study utilized a mixed-methods approach to evaluate the engagement and impact of a digital mental health intervention (Wysa) provided as part of an accessible public health service during COVID-19 in Singapore. The results indicated that users who used the Wysa app following the use of a brief intervention on mindline.sg demonstrated increased engagement with the Wysa app (*P* < 0.001). Among the users who utilized cognitive restructuring on the Wysa app, 91.6% were able to successfully restructure a negative thought. The logistic regression to find any association between changes in negative thought attributions, and restructuring success showed that future-oriented negative thoughts, and internal, stable and global negative thoughts were restructured in a single conversation with Wysa (*P* < 0.001), while other attributional styles required further support.

This analysis evidenced high rates of utilization and engagement across the brief intervention (website) and on the longitudinal intervention (app), compared to the average period seen in digital health (36). In support of previous research, the analysis also evidenced the increase in engagement and retention with a longitudinal intervention, when introduced with a brief intervention. The relationship between the utilization of the brief intervention and the longitudinal intervention is in line with previous research in this domain (23, 24). This suggests that brief interventions can lead to more sustained engagement in mental health support (37). This has the potential to help public health initiatives creating strategies to increase population-level engagement in mental health services.

The stress-diathesis model (38) explains the role of COVID-19 as a stressor in triggering underlying negative thoughts and mental health symptomatology. Most of the negative thoughts being managed by the users were not directly related to the pandemic, but activated by the environmental stressors that the situation amplified. The responses to a mental health application documented the mental and emotional stressors being experienced during the COVID-19 pandemic where digital health applications became a method not only to detect invisible distress, but also to alleviate it (39, 40).

The observed themes within negative thoughts reflected collectivistic values and rules of the culture, where a sense of duty and achievement were emphasized upon (41). The impact of cultural context was visible in the stressors mentioned, and the resolution found reflecting themselves more deeply across the automatic thoughts and intermediate beliefs (42). The core beliefs reflected the theoretical underpinnings of the cognitive model, and demonstrated a universal convergence (43).

In support of prior findings, future-oriented negative thoughts and internal, stable and specific attributional styles revealed a significant likelihood of restructuring with the support of a single AI-led intervention (44). Our findings concur with how cognitive biases contributing to depression (thoughts related to the self or world) exhibit greater rigidity compared to those associated with anxiety (future-oriented thoughts), which were more reformable (45). Similarly, our findings support prior research that acknowledges that the malleability of certain attributional styles (indicating a significant likelihood of a transition between stable to unstable, and global to specific) is associated with positive restructuring, while some also restructured their thought without a change in their attributional style (46, 47). These findings provide useful input into the architecture of digital mental health interventions to improve their utility for populations, and increase their effectiveness.

Transformations of negative attributions toward a more positive valence or reduced self-blame, pervasiveness, and permanence are significant predictors of improved mental health, highlighting the effectiveness of the provided psychological interventions within this study (48–50).

### Strengths and limitations

This study is on a population-level sample from Singapore, which enhances the generalizability of the findings. Similarities in engagement and retention rates between the brief intervention and the app further underscores its strengths. Another strength of this research lies in the utilization of both quantitative and qualitative analyses to examine the efficacy of the digital intervention. Furthermore, this is one of the few studies which examined the efficacy in relation to specific kinds of deconstructed the specific types of negative thoughts that were more readily restructured through the use of psychological models. The examination of negative thoughts revealed major core beliefs, intermediate thoughts, and negative automatic thoughts (NATs) through a cultural lens. These studies enable us to understand the cultural translations and differences in the stressors across a population, and the ability of a brief intervention in managing their distress. This study also contributes to the understanding of attribution styles in cognitive restructuring.

A limitation of this study is that the data was analyzed retrospectively. The researchers also did not know if the users sampled within the study have contracted COVID-19 and its immediate impact on their mental health. This data was also separated from mental health screening data which could have aided analysis.

In the future, studies can examine the relationship between mental health symptoms, negative thought patterns, and the efficacy of digital mental health interventions across both. This study has also utilized the iCHECK-DH reporting checklist to facilitate future meta-research on the topic (Supplementary 1) (51).

## Conclusion

This study utilized a mixed-methods approach to examining the impact of a digital mental health intervention in supporting a nationwide population during a public health crisis. The data documents the ability to improve retention and engagement through a brief intervention, and their perceived helpfulness. The study also evidences efficacy of the intervention in working through mental health distress through the lens of cognitive distortions and attributional styles.

## Data Availability

The datasets presented in this article are not readily available because these are proprietary datasets. Requests to access the datasets should be directed to chaitali@wysa.io.
